# The activation of c-Jun NH_2_-terminal kinase is required for dihydroartemisinin-induced autophagy in pancreatic cancer cells

**DOI:** 10.1186/1756-9966-33-8

**Published:** 2014-01-18

**Authors:** Guang Jia, Rui Kong, Zhi-Bin Ma, Bing Han, Yong-Wei Wang, Shang-Ha Pan, Ying-Hua Li, Bei Sun

**Affiliations:** 1 Department of Pancreatic and Biliary Surgery; Key Laboratory of Hepatosplenic Surgery, the First Affiliated Hospital of Harbin Medical University, Harbin, China; 2 Department of Gastroenterology, the First Affiliated Hospital of Harbin Medical University, Harbin, China; 3 Department of Hematology, the First Affiliated Hospital of Harbin Medical University, Harbin, China; 4 Department of Pancreatic and Biliary Surgery, the First Affiliated Hospital of Harbin Medical University, 23 You Zheng, Harbin 150001, China

**Keywords:** c-Jun NH_2_-terminal kinase, Beclin 1, Apoptosis, LC3, Autophagy, Pancreatic cancer, Dihydroartemisinin

## Abstract

**Background:**

c-Jun NH2-terminal kinases (JNKs) are strongly activated by a stressful cellular environment, such as chemotherapy and oxidative stress. Autophagy is a protein-degradation system in which double-membrane vacuoles called autophagosomes are formed. The autophagy-related gene Beclin 1 plays a key role in this process. We previously found that autophagy was induced by dihydroartemisinin (DHA) in pancreatic cancer cells. However, little is known about the complex relationship between ROS, JNK activation, autophagy induction, and Beclin 1 expression.

**Methods:**

Cell viability and CCK-8 assays were carried out to determine the cell proliferation; small interfering RNAs (siRNAs) were used to knockdown c-Jun NH_2_-terminal kinases (JNK1/2) genes; western blot was performed to detect the protein expression of LC3, JNK, Beclin 1, caspase 3 and β-actin; production of intracellular ROS was analyzed using FACS flow cytometry; autophagy induction was confirmed by electron microscopy.

**Results:**

In the present study, we explored the role of DHA and Beclin 1 expression in autophagy. DHA-treated cells showed autophagy characteristics, and DHA also activated the JNK pathway and up-regulated the expression of Beclin 1. Conversely, blocking JNK signaling inhibited Beclin 1 up-regulation. JNK activation was found to primarily depend on reactive oxygen species (ROS) resulting from the DHA treatment. Moreover, JNK pathway inhibition and Beclin 1 silencing prevented the induction of DHA-induced autophagy.

**Conclusions:**

These results suggest that the induction of autophagy by DHA is required for JNK-mediated Beclin 1 expression.

## Background

c-Jun NH_2_-terminal kinases (JNKs) are strongly activated by a variety of stressful cellular environments, such as chemotherapy and oxidative stress, and induce growth inhibition or cell death [1,2]. The JNK signaling pathway has also been involved in stress-induced apoptosis [3,4], including neuronal death in models of excitotoxicity and stroke [5-7]. JNK is a ‘stress-activated protein kinase’ and plays a pivotal role in both inflammation and cell death [8], with the JNK-induced apoptotic response being mediated, in part, by the expression and/or phosphorylation of proteins belonging to the Bcl-2-related family [9-12]. JNK have a number of targets, including the transcription factor c-Jun, the forkhead transcription factor, and other pro- or anti-apoptotic factors, such as Bax and Bcl-2 [13,14].

Autophagy is a lysosomal pathway involved in the degradation of cytoplasmic macromolecules (such as proteins), and organelles. This process was well preserved during evolution. Although autophagy became a very seductive topic in cancer treatment research, the current literature about autophagy is very confusing due to the association of autophagy with both cell survival and death. Some studies demonstrated that autophagy is induced by stressful conditions, such as metabolic stress, energy need, and chemotherapy [15,16]. Furthermore, several recent reports indicated that reactive oxygen species (ROS) induced autophagy in response to chemotherapy [17,18]. Studies also showed that autophagy promoted cancer cell survival through the generation of metabolic substrates maintaining cellular activity, thereby limiting chemotherapy cytotoxicity [19]. However, the role of autophagy in the efficacy of anti-cancer drugs remains to be defined. Accordingly, this study aimed to further elucidate the role of treatment-induced autophagy in pancreatic cancer cells.

Beclin 1 (the ortholog of yeast Atg6) was the first mammalian autophagy protein to be identified [20], and is a haplo-insufficient tumor suppressor gene. Its gene is frequently mono-allelically deleted in sporadic cancers affecting the prostate, ovaries and breast [21]. Beclin 1 could play a role in recruiting cytosolic proteins for autophagic degradation, or by supplying the autophagosomes with membrane components [22]. Beclin 1 is a member of a Class III PI3K complex involved in autophagosome formation. It mediates the localization of the other proteins involved in autophagy to the pre-autophagosomal membrane [22]. Beclin 1 is also a key factor determining the autophagic or apoptotic fate of cells [23]. Beclin 1 interacts with members of the anti-apoptotic Bcl-2 family via its BH3 domain; Interacting with Bcl-2 proteins competitively inhibits pre-autophagosomal structure formation, thereby inhibiting autophagy [24].

Artemisinin extracted from Artemisia annua, a Chinese medicinal herb, is extremely effective against malaria, with only a few adverse effects. Dihydroartemisinin (DHA) is synthesized from artemisinin. It is more soluble in water, and it is also more effective against malaria than artemisinin. More interestingly, it has also been found to be an effective anti-cancer drug [25-28]. Furthermore, it has been showed that DHA inhibited cell growth and induced apoptosis in pancreatic cancer cells, and that this effect was dose- and time-dependent. Artemisinin has been shown to contain an endoperoxide bridge, which reacts with iron to form ROS. Interestingly, we observed that DHA also activates autophagy in pancreatic cancer cells, and various findings indicate that a number of antineoplastic therapies induce a type of protective, pro-survival autophagy [29-31]. Moreover, ROS-mediated JNK activation is required for the formation of autophagosomes [32]. However, the mechanism by which JNK induces autophagy and the association with anticancer therapy remains mostly unknown.

Therefore, in this present study, we explored the involvement of JNK activation and Beclin 1 expression in DHA-induced autophagy. The aim of the present study was to assess the exact relationships between Beclin 1 expression, JNK pathway activation, and autophagy. We demonstrated that DHA-induced autophagy involved the JNK pathway in pancreatic cancer cell lines, resulting in increased expression of Beclin 1. SP600125 or small interfering RNAs (siRNAs) targeting JNK1/2 inhibited the up-regulation of Beclin 1, as well as autophagy. Results from the present study provide further clues explaining Beclin 1 regulation in autophagy induced by cancer treatments.

## Materials and methods

### Cell lines and culture

The human pancreatic cancer cell lines BxPC-3 (CRL-1687) and PANC-1 (CRL-1469) were purchased from the American Type Culture Collection (ATCC, Manassas, USA). The cells were cultured in RPMI 1640 (SH30809.01B, Hyclone, Thermo Fisher Scientific, Waltham, MA, USA) supplemented with 10% fetal bovine serum (16000, GIBCO, Invitrogen Inc., Carlsbad, CA, USA), 100 units/mL penicillin and 100 μg/mL streptomycin (Invitrogen, 15070–063). Cells were maintained at 37°C in a humidified atmosphere containing 5% CO_2_.

### Reagents and antibodies

The following reagents were purchased from Sigma-Aldrich (St-Louis, MO, USA): DHA (D7439), NAC (A7250), 3MA (M9281), rapamycin (R0395), and SP600125 (S5567). The following antibodies were purchased from Santa Cruz Biotechnology (Santa Cruz, CA, USA): JNK (sc-7345), p-JNK (sc-6254), and β-actin (sc-130301). The following antibodies were purchased from Cell Signaling Technology (Danvers, MA, USA): caspase-3 (9665), LC3 (2775), and Beclin 1 (3738).

### Cell proliferation assay

Cells were plated in 96-well or 6-well cell culture plates (5 × 10^3^ cells per well) and treated with various compounds, as indicated in the figure legends. At the end of treatments, cell proliferation was evaluated using a Cell Counting Kit-8 (CCK-8, CK04-13, Dojindo Molecular Technologies, Kimamoto, Japan) or Crystal Violet (C6158, Sigma) staining according to the instructions of the manufacturer, or by photometric measurements to determine cell viability. Three or four independent experiments were performed for each assay condition.

### Electron microscopy

Cells were harvested by trypsinization, fixed in 2.5% glutaraldehyde/4% paraformaldehyde in 0.1 mol/L cacodylate buffer and then postfixed in 1% osmium tetroxide buffer. After dehydration in acetone, cells were embedded in spur resin, and thin sections (90 nm) were cut using a Reichert Ultracut E microtome. The sectioned grids were stained with a saturated solution of uranyl acetate and lead citrate. Sections were examined at 80 kV using a JEOL 1200EX transmission electron microscope.

### Western blot analysis

Cells were pelleted at 500 g for 5 min and lysed in cold lysis buffer [20 mmol/L Tris–HCl (pH 7.5), 150 mmol/L NaCl, 1 mmol/L EDTA, 1 mmol/L EGTA, 1% Triton X-100, 2.5 mmol/L sodium PPi, 1 mmol/L β-glycerolphosphate, 1 mmol/L Na_3_VO_4_, 1 μg/mL leupeptin, and 1 mmol/L phenylmethylsulfonyl fluoride]. After sonication for 5 s, lysates were clarified by centrifugation at 12,000 g for 30 min at 4°C. Identical amounts (25 μg of protein) of cell lysates were separated by 8% or 15% SDS-PAGE gel electrophoresis, and the proteins were transferred onto nitrocellulose or polyvinylidene difluoride membranes. Membranes were then incubated in a blocking solution consisting of 5% powered milk in TBST [10 mmol/L Tris–HCl (pH 8.0), 150 mmol/L NaCl, and 0.1% Tween 20] for 1 h, followed by immunoblotting with the respective antibodies. The proteins of interest were detected using enzyme-linked chemiluminescence, according to the manufacturer’s protocol.

### Transfection of siRNA

The target sequence for the JNK1/2-specific siRNA was 5’-AAA AAG AAU GUC CUA CCU UCU-3’ (GeneBank accession number NM002750.2), the target sequence for the Beclin 1-specific siRNA was 5’-UGG AAU GGA AUG AGA UUA ATT-3’ (GeneBank accession number NM003766.2) and the target sequence for the Atg-5-specific siRNA was 5’-TGT GAT GTT CCA AGG AAG AGC-3’ (GeneBank accession number NM004849.2). The control siRNAs (no silencing) for these siRNAs were synthesized by GenePharma Co. (Shanghai, China). siRNAs were transfected into the cells using Lipofectamine 2000 (Invitrogen) according to the protocol provided by the manufacturer.

### Determination of intracellular ROS production

Production of intracellular ROS was measured using the fluorescent dye 2,7-dichlorofluorescein diacetate (DCF-DA). The cells were plated at a density of 1 × 10^5^ in 6-well plates, allowed to attach overnight, and exposed to the treatments described in the figure legends. The cells were then incubated with 10 M DCFHDA for 20 min at 37°C in a 5% CO_2_ incubator, washed and resuspended in PBS at 1 × 10^6^ cells/ml. The cells were analyzed by FACS flow cytometry at an excitation wavelength of 514 nm, and the fluorescence intensity of DCF was measured at an emission wavelength of 525 nm. Untreated cells served as controls. The amount of intracellular ROS was expressed as the fold-increase of DCF fluorescence compared with the control.

### Analysis of autophagy by GFP-LC3 redistribution

To monitor the formation of GFP-LC3 puncta, the cells were transiently transfected with 1.0 mg GFP-LC3 plasmid, and then treated as described in the figure legends. After treatment, autophagy was measured by light microscopic quantification of cells transfected with GFP-LC3, as previously described [33].

### Statistical analysis

Results are expressed as mean ± SD. Statistical analysis was performed using the Student’s t test, with *P* < 0.05 deemed as statistically significant. All experiments were repeated at least three times.

## Results

### DHA possesses cytotoxic effects on pancreatic cancer cells

**Figure 1:**
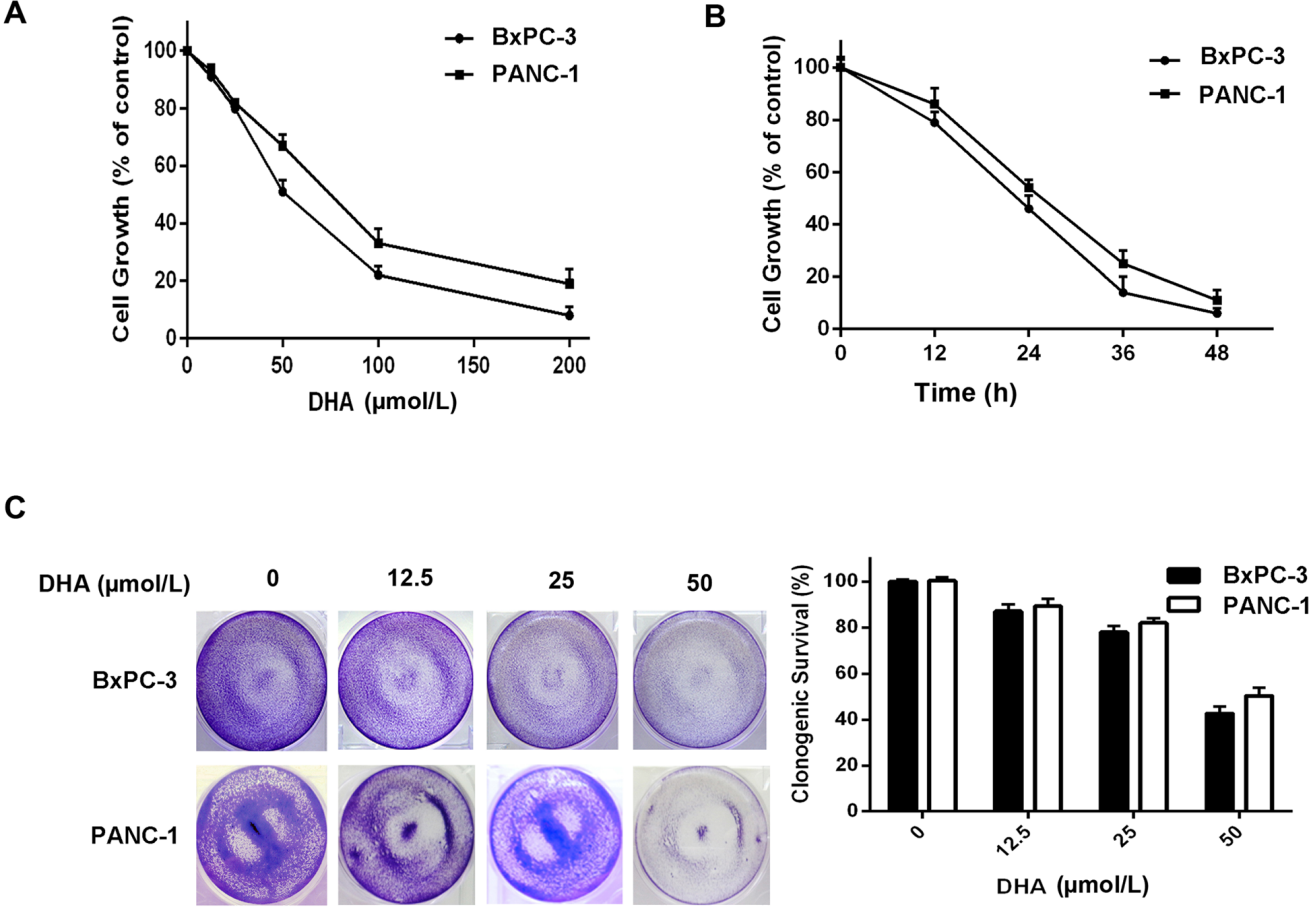
**Cell death induced by DHA in pancreatic cancer cells. (A, B)** BxPC-3 and PANC-1 cells were treated with different concentrations of DHA for 24 h, or treated with 50 μmol/L DHA for different times. The percentage of cell death was determined by a CCK-8 assay. **(C)** BxPC-3 and PANC-1 cells were treated with different concentrations of DHA for 24 h and washed with PBS. Cells were then incubated for an additional 7 d and stained with crystal violet, as described in the Materials and methods section.

DHA is cytotoxic for a variety of types of cancer cells, while essentially having no effect in normal cells [25-28]. To determine DHA effects on pancreatic cancer cells, we treated BxPC-3 and PANC-1 human pancreatic cancer cells with different concentrations of DHA for 24 h. This treatment was followed by a cell proliferation and cytotoxicity assay (CCK-8) to assess cell viability. DHA significantly inhibited the growth of the pancreatic cancer cells, and DHA cytotoxicity in these cells was dose- and time-dependent (Figure 1A and B). We used a clonogenic assay to confirm the effects of DHA on these cell lines and to determine whether DHA affected long-term colony formation; the number of surviving colonies was also markedly inhibited (Figure 1C). These results indicate that DHA has a specific effect on human pancreatic cancer cell lines.

### Treatment with DHA induces caspase-3-dependent cell death and autophagy in pancreatic cancer cells

**Figure 2:**
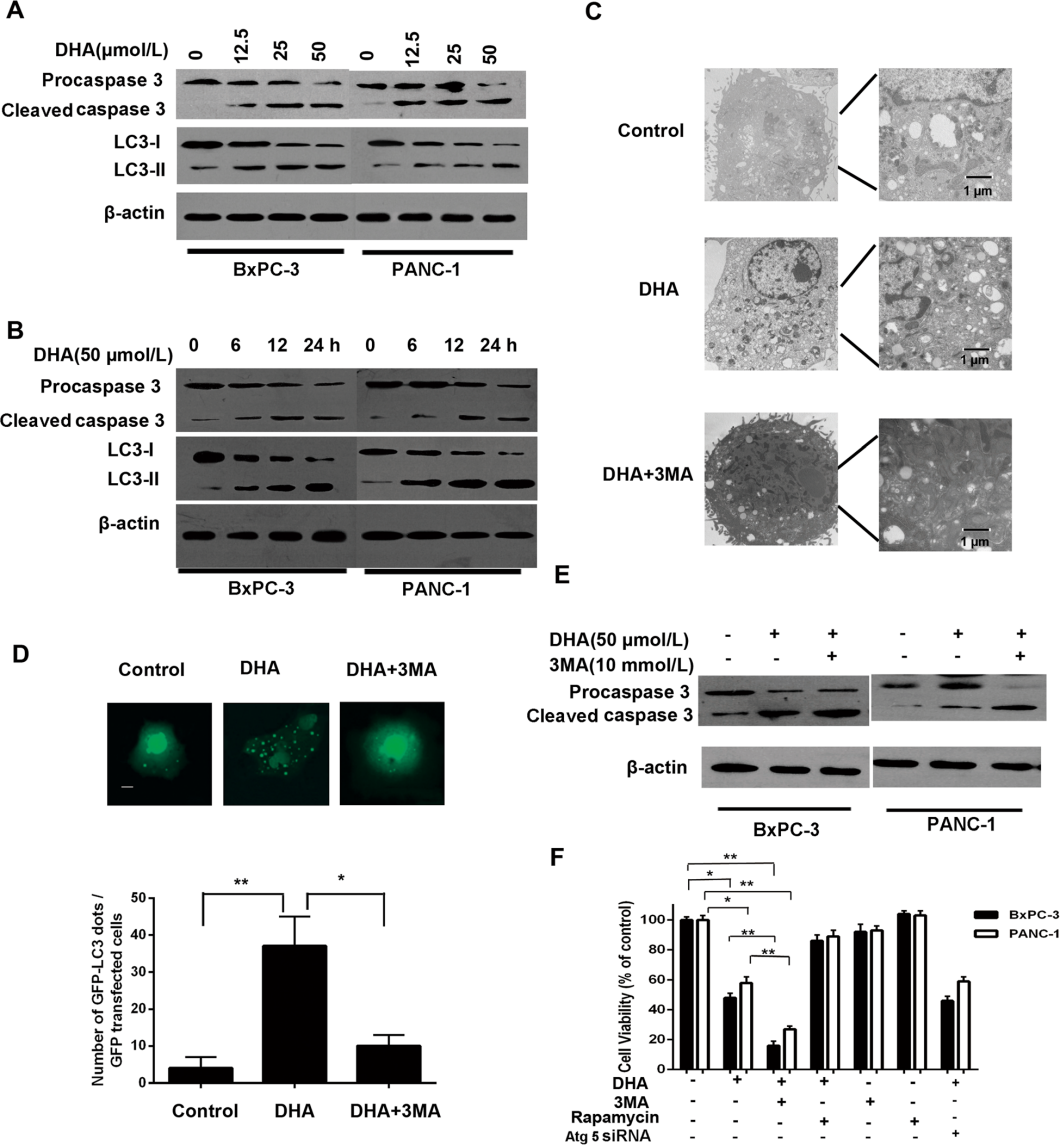
**DHA triggers apoptosis and autophagy in pancreatic cancer cells. (A, B, E)** Immunoblot analysis of LC3 and caspase-3 levels in BxPC-3 and PANC-1 cell lines treated with different concentrations of DHA for 24 h, or treated with 50 μmol/L DHA for different times in the presence or absence of 10 mmol/L 3MA. **(C)** Representative electron micrographs of BxPC-3 cells treated with 50 μmol/L DHA for 24 h in the presence or absence of 10 mmol/L 3MA. **(D)** Top, representative images of GFP-LC3 staining in BxPC-3 cells transfected with the GFP-LC3 plasmid, followed by 50 μmol/L DHA for 24 h with or without 3MA (10 mmol/L); bottom, number of GFP-LC3 dots scored in 100 transfected cells. Bar: 5 μm. **(F)** BxPC-3 and PANC-1 cells were treated with 50 μmol/L DHA for 24 h in the presence or absence of 10 mmol/L 3MA or 1 μmol/L rapamycin or Atg 5 siRNA. The percentage of dead cells was determined by a CCK-8 assay. *P < 0.05; **P < 0.01.

To determine if apoptosis depends on caspase-3, we first assessed caspase-3 cleavage, an essential step in the caspase pathway. A western blot analysis in DHA-treated cells revealed decreased procaspase-3 levels, and increased levels of the cleaved, active forms (Figure 2A). Following DHA treatment, we detected caspase-3 cleavage in the two cancer cell lines for all concentrations and time (Figure 2A and B).

We next determined whether DHA treatment induced autophagy in tumor cells. The autophagy marker LC3-II, a cleaved and then conjugated to phosphatidylethanolamine product of microtubule-associated protein 1 light chain 3, was assessed in an immunoblotting assay. After DHA treatment, LC3-II was dose- and time-dependently increased in BxPC-3 and PANC-1 cells (Figure 2A and B). Autophagy induction by DHA was confirmed by electron microscopy and a GFP-LC3 cleavage assay, which showed abundant double-membrane vacuoles (Figure 2C) and an increased number of cells with GFP-LC3 punctae (Figure 2D) in the cytoplasm of DHA-treated cells. In contrast, these vacuoles were rarely observed in vehicle-treated pancreatic cancer cells (Figure 2C).

To evaluate the role of DHA-induced autophagy, we treated cells with 3MA, an inhibitor of autophagy, to further decrease autophagy in the pancreatic cancer cells during DHA treatment. The inhibition of DHA-induced autophagy by 3MA significantly increased the expression of cleaved caspase-3 (Figure 2E).

To further confirm whether autophagy protected the pancreatic cancer cells from DHA-induced apoptosis, the effect of 3MA (an autophagy inhibitor) and rapamycin (an autophagy activator) on DHA-induced cell death was examined. Autophagy inhibition significantly increased the incidence of cell death, whereas autophagy activation decreased cell death, as assessed by a CCK-8 assay (Figure 2F). Additionally, we also found that knockdown of Atg5 did not change the effect of DHA on cell viability (Figure 2F). These findings indicate that DHA induced some kind of protective, pro-survival autophagy increasing the resistance of the cancer cells against DHA therapy. The induction of autophagy was independent on Atg5. This increase in cell death via autophagy inhibition would lead to the inhibition of tumor growth.

### Treatment with DHA activates JNK and beclin 1 in pancreatic cancer cells

**Figure 3:**
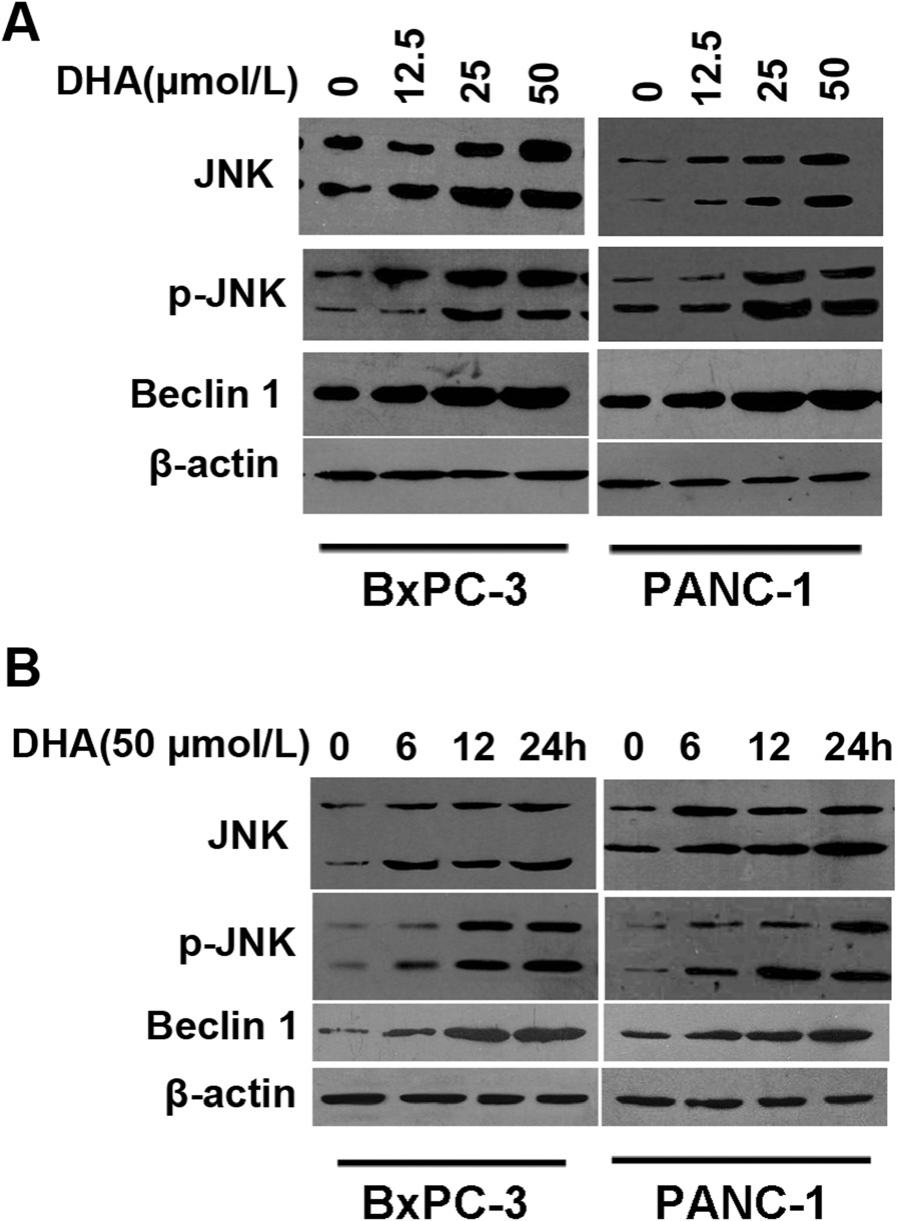
**The effect of DHA on JNK phosphorylation and the up**-**regulation of Beclin 1 expression in pancreatic cancer cells. (A, B)** BxPC-3 and PANC-1 cells were treated with various concentrations of DHA for 24 h or with 50 μmol/L DHA for different times. The expression levels of JNK, phospho-JNK, and Beclin 1 protein were analyzed by immunoblotting **(A)**. After treatment with DHA for different times, cell lysates were analyzed by immunoblotting using antibodies against JNK, phospho-JNK, and Beclin 1 **(B)**.

DHA activates mitogen-activated protein kinase (MAPK) signaling pathways in a number of cell types. To study the MAPK/JNK signaling pathway in DHA-induced autophagy, we first measured JNK activation by DHA. DHA stimulated JNK phosphorylation in a dose- and time-dependent manner in the two cell lines (Figure 3A).

The induction of autophagy by DHA was confirmed previously. To determine if DHA upregulated Beclin 1 expression in BxPC-3 and PANC-1 cells, Beclin 1 protein expression was measured. Immunoblotting revealed dose- and time-dependent increases in Beclin 1 expression in cells exposed to DHA (Figure 3B). These findings demonstrated that treatment with DHA activates JNK and Beclin 1 in both pancreatic cancer cell lines in a dose- and time-dependent manner.

### Up-regulation of JNK expression following DHA treatment depends on ROS

**Figure 4:**
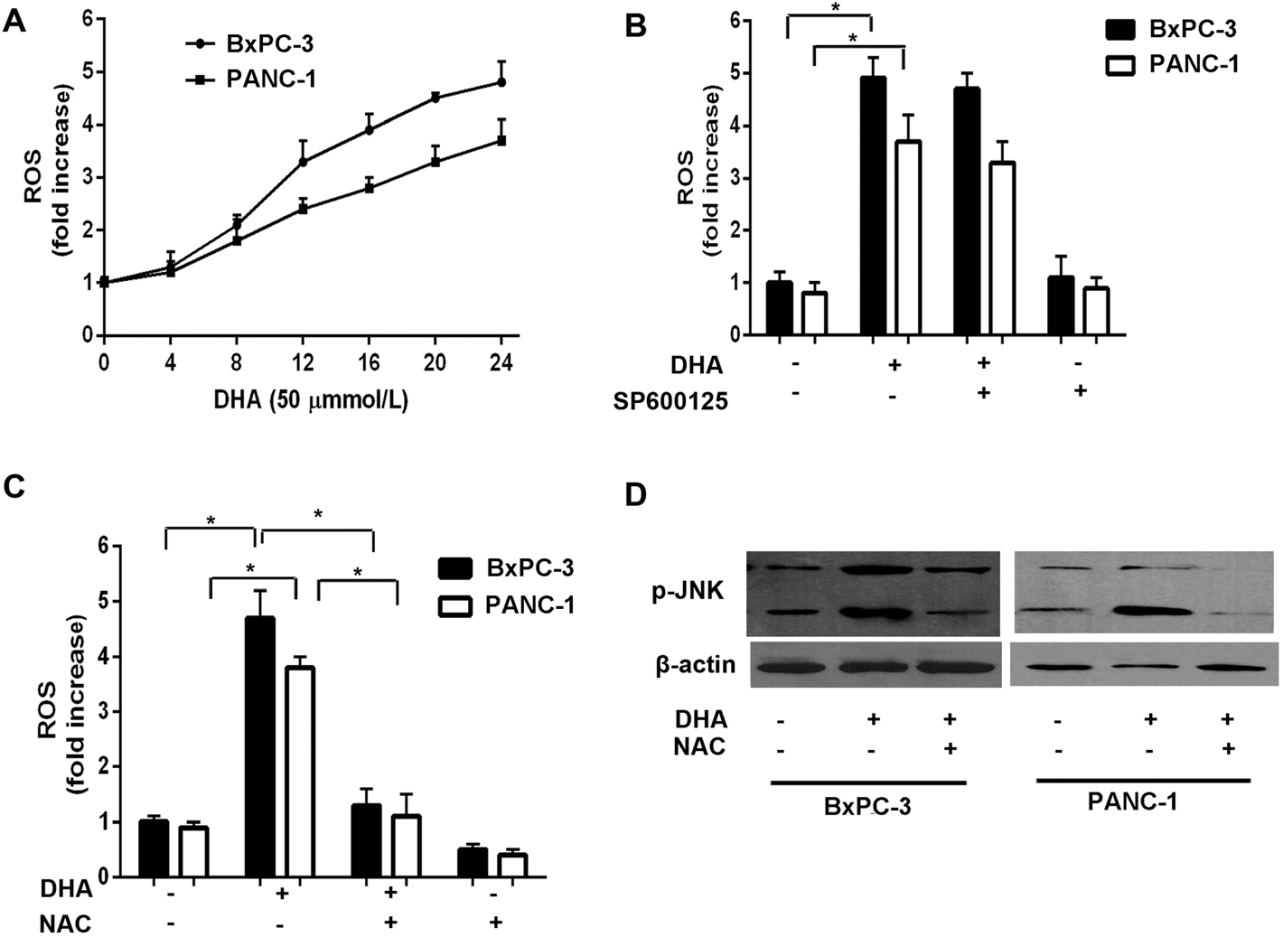
**JNK expression induced by DHA is dependent on ROS generation. (A)** BxPC-3 and PANC-1 cells were treated with 50 μmol/L DHA for different times, and then subjected to flow cytometry to measure ROS levels, as described in the Materials and Methods section. **(B, C)** BxPC-3 and PANC-1 cells were treated with 50 μmol/L DHA for 24 h in the presence or absence of 10 μmol/L SP600125 or 10 mmol/L NAC pretreatment for 1 h and then subjected to flow cytometry to measure the levels of ROS. **(D)** immunoblot analysis of the phospho-JNK levels in BxPC-3 and PANC-1 cells treated with the indicated concentrations of DHA for 24 h in the presence or absence of 10 mmol/L NAC. *P < 0.05.

JNK pathway over-activation is crucial to many processes leading to cell death, including chronic and acute oxidative stress. Although ROS can increase JNK signaling via the activation of upstream kinases or the inactivation of phosphatases, other unknown mechanisms are likely to contribute to ROS-induced JNK increases in pancreatic cancer cells. To exclude the possibility that other mechanisms were responsible for our observations, we measured ROS levels in response to DHA. ROS were increased after DHA treatment and did not differ between the two tested cell lines (Figure 4A).

To further determine whether DHA treatment requires JNK activation to generate ROS, we pre-treated BxPC-3 cells with SP600125 (a specific JNK inhibitor) for 1 h, before exposing them to DHA. In contrast to DHA treatment alone, SP600125 pretreatment prevented alterations in ROS levels (Figure 4B). To examine whether ROS inhibition impacted JNK signaling, we compared JNK activation with or without N-acetyl-L-cysteine (NAC, a ROS inhibitor). NAC pretreatment significantly lowered intracellular ROS compared with DHA-treated cells (Figure 4C). More importantly, the degree of JNK activation after DHA treatment was decreased in the cells pretreated with NAC (Figure 4D), and this decreased JNK activation was related to the inhibition of ROS formation. These results indicate that JNK expression following DHA treatment depends on ROS.

### Inhibition of JNK expression down-regulates beclin 1 and reduces autophagy

**Figure 5:**
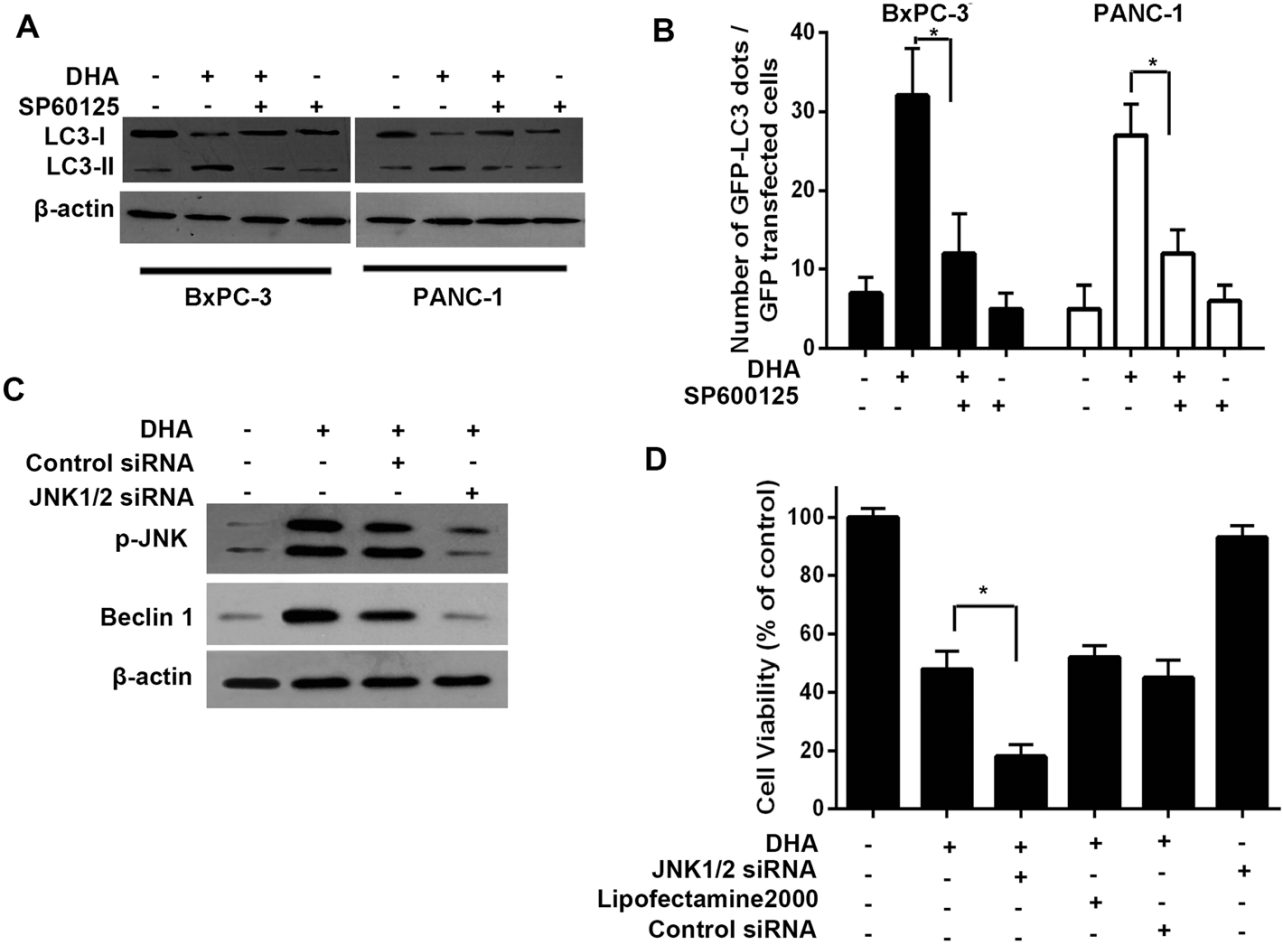
**Inhibition of JNK expression downregulates Beclin 1 and represses autophagy. (A)** BxPC-3 and PANC-1 cells were treated with different concentration of DHA for 24 h in the presence or absence of 10 μmol/L SP600125 pretreatment for 1 h. The expression levels of the LC3-I and LC3-II proteins were subsequently analyzed by immunoblotting. **(B)** BxPC-3 cells transfected with the GFP-LC3 plasmid, followed by 50 μmol/L DHA for 24 h with or without SP600125 (10 μmol/L). The number of GFP-LC3 dots was subsequently scored in 100 transfected cells. **(C)** BxPC-3 cells were treated with 50 μmol/L DHA for 24 h in the absence or presence of JNK1/2 siRNA. The expression levels of phospho-JNK and Beclin 1 protein were subsequently analyzed by immunoblotting. **(D)** BxPC-3 cells transfected with a non-targeting RNA or a JNK1/2-targeted siRNA were treated with 50 μmol/L DHA for 24 h. At the end of the treatment, cell viability was measured using a CCK-8 assay. *P < 0.05.

To further assess the role of JNK in DHA-induced autophagy, cells were pretreated with SP600125 (10 mM) for 1 h, and were then exposed to DHA. In contrast to DHA alone, SP600125 pretreatment blocked the increase in LC3-II induced by DHA (Figure 5A). Furthermore, SP600125 treatment decreased the punctate foci of LC3 in the cytoplasm (Figure 5B).

To determine if JNK activation is required for Beclin 1 expression in the context of DHA-induced autophagy, JNK expression was knocked-down using a siRNA directed against JNK1/2. siRNA transient transfection down-regulated JNK (Figure 5C). More importantly, siRNA-mediated JNK down-regulation prevented the DHA-induced up-regulation of Beclin 1 protein in addition to efficiently inhibiting the level of JNK phosphorylation in pancreatic cancer cells (Figure 5C). These findings suggest that JNK could be directly involved in the DHA-induced increased Beclin 1 expression.

To test whether blockage of DHA-activated autophagy through JNK inhibition could enhance cytotoxicity, tumor cells were transfected with a non-targeting RNA or a siRNA targeting JNK, and were then exposed to DHA. DHA cytotoxicity was significantly increased by silencing the expression of JNK in these cells (Figure 5D). Taken together, these findings indicate that JNK could be directly involved in the DHA-induced increased Beclin 1 expression. Furthermore, it can be concluded that the inhibition of JNK could enhance the efficacy of DHA by inhibiting autophagy.

### Beclin 1 siRNA knock-down blocks DHA-induced autophagy

**Figure 6:**
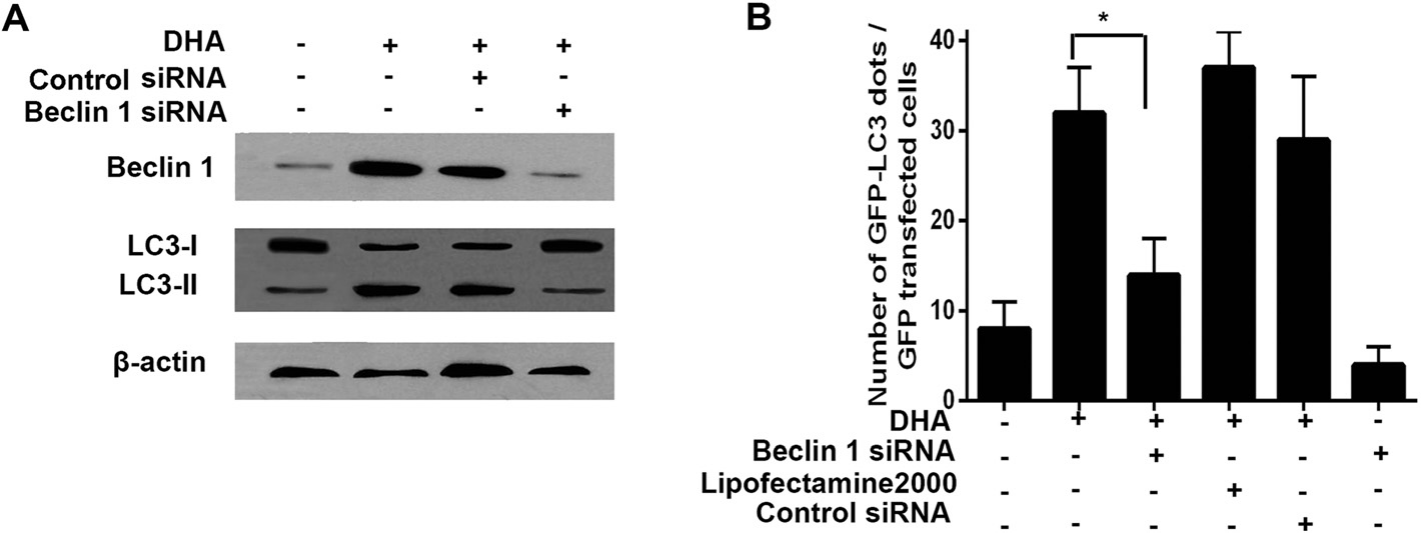
**Beclin 1 is required for DHA**-**induced autophagy. (A)** BxPC-3 cells transfected with a non-targeting RNA or a Beclin 1-targeted siRNA were treated with 50 μmol/L DHA for 24 h. At the end of treatment, the expression levels of the Beclin 1, LC3-I, and LC3-II protein were analyzed by immunoblotting. **(B)** BxPC-3 cells transfected with the GFP-LC3 plasmid, followed by 50 μmol/L DHA for 24 h in the absence or presence of Beclin 1 siRNA. The number of GFP-LC3 dots was subsequently scored in 100 transfected cells. *P < 0.05.

To potentially use the intrinsic role of Beclin 1 in DHA-induced autophagy, we investigated the effects of Beclin 1 knock-down on DHA-induced apoptosis. We designed siRNAs down-regulating Beclin 1 expression. Beclin 1 silencing significantly inhibited LC3-II induction by DHA (Figure 6A). Fewer Beclin 1-silenced cells exhibited GFP-LC3 punctae compared to the control DHA- and siRNA-treated cells (Figure 6B). These results suggest that Beclin 1 could play a crucial role in DHA-induced autophagy.

## Discussion

The association between apoptosis and autophagy remains controversial. Experimental evidences suggest that autophagy can mediate apoptosis, and that autophagy would be one of the three forms of cell death, together with apoptosis and necrosis [34]. However, several studies demonstrated that autophagy would also be critical for cell survival [35-37]. Our research group has extensively studied the effect of the anticancer agent DHA on pancreatic cancer cells, and we showed that DHA significantly inhibited cell growth and induced apoptosis in pancreatic cancer cells [38]. Interestingly, DHA treatment also induces autophagy in pancreatic cancer cells. Therefore, in the present study, we explored the role of autophagy induced by DHA and its mechanisms in pancreatic cancer cells.

Autophagy may be used by some cancer cells types as a mean to adapt to the stressful environment observed within solid tumors (i.e. hypoxic, nutrient-limiting, and metabolically stressful), as well as in artificial conditions induced by cytotoxic agents [39]. Studies in human cancer cell lines showed that a number of anticancer therapy modalities, including radiations and chemotherapy induced autophagy as a protective mechanism aiming toward survival [30,31]. Moreover, in cancer cell lines, inhibition of autophagy may be a therapeutic target under some circumstances. Indeed, inhibiting autophagy has been shown to enhance cancer cells’ therapies such as DNA-damaging agents, hormone therapies for breast and ovarian cancer, and radiations [40-43]. In the present study, we used 3MA (an autophagy inhibitor) to inhibit DHA-induced autophagy and rapamycin (an autophagy activator) to enhance it. The data clearly demonstrated that DHA can induce autophagy and that inhibition of autophagy can enhance the sensitivity of pancreatic cancer cells to DHA. These findings showed that DHA therapy induced a kind of protective autophagy in pancreatic cancer cells, increasing their resistance to DHA and hence their survival, and that inhibiting autophagy may led to increased apoptosis. Such enhanced apoptosis should normally reduce tumor growth.

The excessive production of ROS can overcome cells’ defenses against ROS, thus leading to oxidative stress, which is involved in cell injury and apoptosis. Studies showed that DHA led to ROS generation in papilloma virus-expressing cell lines, inducing oxidative stress and, ultimately, apoptosis [25]. Recent studies in models of hepatocyte oxidative stress emphasized that the superoxide generator menadione mediated the activation of MAPK/JNK and c-Jun [44,45]. ROS is known to increase JNK by activating upstream kinases or by inactivating phosphatases, but other unknown mechanisms might contribute to DHA- and ROS-induced increases in JNK. In our study, we confirmed that the up-regulation of JNK expression following DHA treatment depended on ROS. Accordingly, several studies demonstrated that JNK pathway over-activation is crucial to the different forms of hepatocyte apoptosis, including the forms induced by chronic and acute stress from ROS [46,47]. Therefore, we conclude that the generation of ROS also contributes to JNK activation following DHA treatment.

The resolution of the function of JNK in autophagy regulation is imminent. It was observed that autophagy associated with endoplasmic reticulum stress (ERS) was inhibited in IRE1-deficient cells or in cells treated with a JNK inhibitor, suggesting that IRE1-JNK is required for ERS-induced autophagy [32]. These data suggest that JNK may play a crucial role in autophagy. In this study, we showed that DHA activated the JNK pathway and mediated autophagy. We showed that DHA increased JNK phosphorylation in pancreatic cancer cells in a time- and dose-dependent manner. Activation of the JNK pathway results in Bcl-2 phosphorylation, an event known to enhance autophagy by disrupting the Bcl-2/Beclin 1 competitive interaction [33]. Bcl-2 is able to regulate Beclin 1-induced autophagy by directly binding to Beclin 1, and thus preventing its activation [48]. Similarly, we observed that JNK was involved in Beclin 1 expression, which then played a crucial role in protective autophagy in DHA-induced cancer cells. Although, Beclin 1 up-regulation by JNK was observed after autophagy induced by the anticancer drug topotecan, the data presented in the present study constitute the first evidence that Beclin 1 is regulated by JNK in pancreatic cancer cells.

## Conclusions

Our results suggest that autophagy was induced by DHA in the studied human pancreatic cancer cell lines. DHA also activated JNK, thus up-regulating Beclin 1. JNK activation primarily depends on ROS, which is generated by DHA treatment. Moreover, inhibiting the JNK pathway and silencing Beclin 1 expression could inhibit DHA-induced autophagy. These results suggest that autophagy can be induced by DHA through Beclin 1 expression induced by JNK. Silencing of JNK kinase may constitute appealing therapeutic target for a generalized strategy to treat cancer through blunting of autophagy. This may support a novel therapeutic strategy against pancreatic cancer in clinical settings.

## Abbreviations

JNK: c-Jun NH_2_-terminal kinase; DHA: Dihydroartemisinin; ROS: Reactive oxygen species; siRNAs: Small interfering RNAs; 3MA: 3-methyladenine; MAPK: Mitogen-activated protein kinase; NAC: N-acetyl-L-cysteine.

## Competing interests

The authors declare that they have no competing interests.

## Authors’ contributions

GJ: study concept and design, experimental work and acquisition of data, drafting of the manuscript, analysis and interpretation of data. RK, ZBM, BH: experimental work and acquisition of data. YWW, SHP: analysis and interpretation of data. YHL, BS: study concept and design, analysis and interpretation of data, critical revision of the manuscript for important intellectual content of the manuscript. All authors read and approved the final manuscript.
